# Multidimensional characterization of the conical intersection seam in the normal mode space†
†Electronic supplementary information (ESI) available: SEVI (slow electron velocity map imaging) spectra *via* selected S_1_ vibrations, (two- or three-component) deconvolution of TKER distributions of two thioanisole isotopomers. See DOI: 10.1039/d0sc02045a


**DOI:** 10.1039/d0sc02045a

**Published:** 2020-06-16

**Authors:** Heesung Lee, So-Yeon Kim, Sang Kyu Kim

**Affiliations:** a Department of Chemistry , KAIST , Daejeon 34141 , Republic of Korea . Email: sangkyukim@kaist.ac.kr

## Abstract

Multidimensional conical intersection seam has been characterized by utilizing the dynamic resonances in the nonadiabatic transition probability experimentally observed in the predissociation of thioanisole isotopomers. The nonadiabatic bifurcation behavior of the reactive flux into either the Herzberg type-I (electronic) or type-II (vibrational) predissociation pathway is found to be strongly dependent on the quantum nature of the S_1_/S_2_ vibronic eigenstate, providing the essential information about structure and dynamic character of the conical intersection seam projected onto the normal mode space. By modifying the nature of the normal mode space through partial or full H/D substitution of the molecule, multiple aspects of the conical intersection seam could be characterized from different viewpoints set by the adjusted normal mode space. Theoretical calculations of potential energy curves along selected normal mode displacements support the experiment.

## Abstract 2

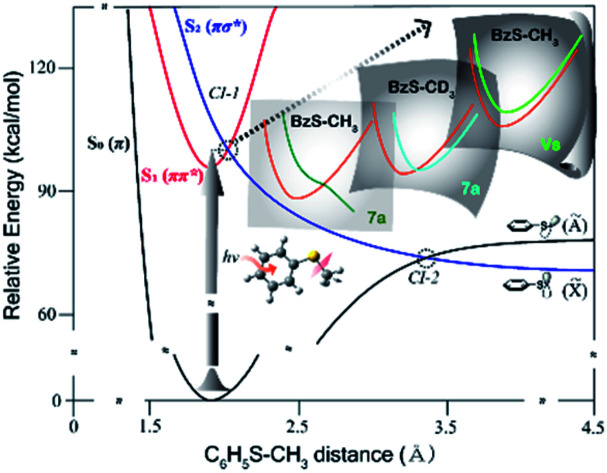
Multidimensional conical intersection seam has been characterized by utilizing the dynamic resonances in the nonadiabatic transition probability experimentally observed in the predissociation of thioanisole isotopomers.

## Introduction

**Fig. 1:**
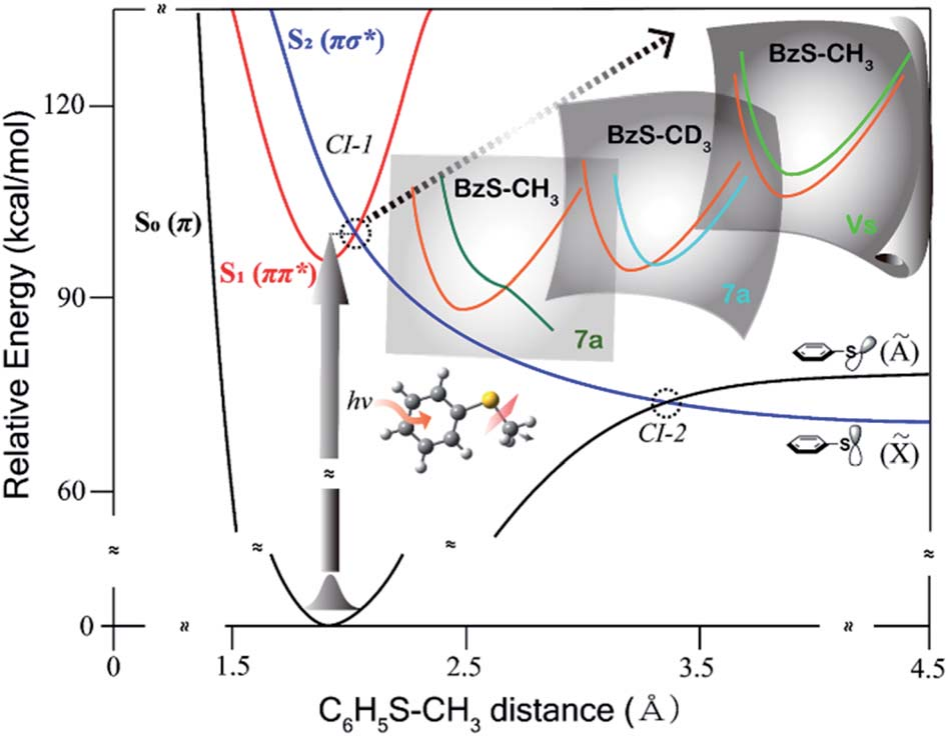
Schematic potential energy curves along the S–CH_3_ bond elongation coordinate. Projections of the conical intersection seam onto the different normal mode spaces are depicted just as approximated cartoons.

Nonadiabatic transition occurs most efficiently when the reactive flux is prepared in the proximity of the conical intersection (CI) seam.^1^,^2^ In the S–CH_3_ bond predissociation reaction of thioanisole (C_6_H_5_SCH_3_), the reactive flux prepared by the excitation to the first electronically excited (S_1_; ππ*) state bifurcates into either the adiabatic or nonadiabatic pathway at the first S_1_/S_2_ conical intersection where the upper-lying S_2_ (πσ*) state is repulsive along the S–CH_3_ bond elongation coordinate.^3^–^5^ The flux sliding on S_2_ then undergoes another bifurcation at the second S_0_/S_2_ conical intersection to give either the excited (Ã) or ground (X[combining tilde]) state of the C_6_H_5_S˙ radical at the asymptotic limit as the former or latter is diabatically correlated from S_0_ or S_2_, respectively, Fig. 1. The nonadiabatic transition efficiency is then reflected in the X[combining tilde]/Ã product branching ratio, giving the quantitative estimation of the otherwise unambiguous nonadiabatic transition probability. Both S_1_/S_2_ and S_0_/S_2_ conical intersections are located at the planar geometry and share the same branching plane in terms of its gradient and coupling vectors.^3^–^5^ Furthermore, as the S_1_/S_2_ conical intersection is correlated to the S_0_/S_2_ conical intersection by the repulsive S_2_ state in the very short S–CH_3_ bond length region, the quantum mechanical character of the initial reactive flux especially along the degrees of freedom orthogonal to the reaction coordinate is not likely to be modified.^3^–^8^ This has provided a great chance to characterize the S_1_/S_2_ conical intersection seam by monitoring the X[combining tilde]/Ã product branching ratio with varying the S_1_ vibronic mode excitation within the Franck–Condon region.

A decade ago, our group reported the experimental result that the X[combining tilde]/Ã branching ratio (*Γ*) shows a striking dynamic resonance upon a specific vibronic mode excitation of thioanisole.^3^ This has provided the unique opportunity to unravel the structure and dynamic property of the conical intersection, as the resonance-like peak in the nonadiabatic transition probability should represent that the reactive flux prepared by the specific vibronic mode excitation resides in the proximity of the conical intersection. The vibronic mode responsible for the dynamic resonance, from the spectroscopic analysis and theoretical calculation, has been identified to be the 7a mode which involves the S–CH_3_ asymmetric stretching.^3^–^13^ This mode should be at least partially parallel to the gradient vector comprising the branching plane of the S_1_/S_2_ conical intersection. The flux prepared by the 7a mode excitation is thus expected to undergo the nonadiabatic transition quite efficiently. Recently, our group has carried out the picosecond time-resolved pump-probe experiment to unravel the temporal behavior of the reactive flux associated with each of many different S_1_ vibronic modes of thioanisole.^5^ Interestingly, it has been found that the reactive flux at the 7a mode excitation bifurcates into the adiabatic or nonadiabatic pathway with quite distinct reaction rates. In the adiabatic channel, the nuclear motion of the reactive flux follows the low-lying adiabatic surface under the S_1_/S_2_ conical intersection and the S_1_ vibrational energy is fully or partially utilized to overcome the multi-dimensional adiabatic reaction barrier. This belongs to the Herzberg type-II vibrational predissociation.^14^,^15^ The adiabatic nature of the flux then persists along the entire reaction pathway, avoiding the curve crossing at the S_0_/S_2_ conical intersection to give the Ã state of the C_6_H_5_S˙ radical rather exclusively at the asymptotic limit. On the other hand, in the nonadiabatic channel, the reactive flux on the upper adiabatic surface above the S_1_/S_2_ conical intersection undergoes the nonadiabatic transition into the low-lying adiabatic surface most efficiently through the conical intersection, and this should belong to the Herzberg type-I electronic predissociation.^14^,^15^ The resultant flux then bifurcates again at the S_0_/S_2_ conical intersection this time with the significant probability of the nonadiabatic transition, as the reactive flux which was nonadiabatically leaked from the first S_1_/S_2_ conical intersection should have been quantum-mechanically shaped for the efficient nonadiabatic passage through the second S_0_/S_2_ conical intersection. The reaction rate on the adiabatic passage (Herzberg type-II) has been found to be much slower than that of the nonadiabatic path (Herzberg type-I). The experimental fact that the nonadiabatic transition probability strongly depends on the initial quantum state of the reactive flux indicates that the structure and energetics of the conical intersection seam could be possibly characterized in the normal mode space over the Franck–Condon region.

In contrast to the fact that the nonadiabatic transition probability is enhanced by the excitation of the 7a mode only for the bare thioanisole, it has been found that the three more vibronic modes in addition to 7a are responsible for dynamic resonances in the partially deuterated thioanisole (C_6_H_5_S–CD_3_).^6^ These modes include ν_S_ (S–CD_3_ symmetric stretching), β (CD_3_ bending), and an unidentified mixed mode. The dynamic resonances at multiple vibronic modes already reflect that the conical intersection is located on the multi-dimensional seam. Moreover, this suggests that the gradient vector comprising the branching plane may be described by the combination of normal modes in terms of their structural changes and associated energetics. What would be the reason why C_6_H_5_S–CD_3_ shows multiple dynamic resonances whereas the only 7a mode is responsible for a dynamic resonance in the undeuterated thioanisole (C_6_H_5_S–CH_3_)? As the electronic structures are expected to be little influenced by the H/D substitution, it is most likely that the structure and electronic property of the S_1_/S_2_ conical intersection seam would be identical for both isotopomers. The subtle differences in the nuclear displacement vectors of the normal modes of C_6_H_5_S–CH_3_ and C_6_H_5_S–CD_3_ should be then responsible for the different behaviors of dynamic resonances in these two isotopomers. In other words, the conical intersection seam associated with the sulfur–methyl bond dissociation has been projected onto different sets of normal mode coordinates for different isotopomers. In this sense, one may then be able to characterize the conical intersection seam in a number of different normal mode spaces subject to subtle changes by the partial or full H/D substitution of the molecule. As a matter of fact, our group reported that dynamic resonances at the 7a or ν_S_ mode excitation are quite robust for the particular thioanisoles of which the methyl moiety was partially deuterated (C_6_H_5_S–CH_2_D, C_6_H_5_S–CHD_2_).^7^,^9^ In this work, for the more complete characterization of the conical intersection, we have carried out the experiment on the fully deuterated thioanisole (C_6_D_5_S–CD_3_) as well as the partially deuterated in total but fully deuterated one on the benzene moiety (C_6_D_5_S–CH_3_). More importantly, we have carried out theoretical calculations on the first (S_1_) and second (S_2_) excited potential energy curves with respect to 7a and ν_S_ normal modes, unravelling the nature of the conical intersection seen from many different viewpoints set by different normal mode spaces of various isotopomers.

## Methods

The sample (C_6_D_5_S–CH_3_; 98.5% and C_6_D_5_S–CD_3_; 97.17%) was purchased (MediGen Co.) and used without further purification. The gas mixture of the sample with He (Ne, Ar) was supersonically expanded through a nozzle orifice and skimmed into the differentially-pumped vacuum chamber with the backing pressure of 1–2 atmosphere at the repetition rate of 10 Hz. Detailed experimental setup had been described in previous works.^7^,^9^,^16^ The UV laser pulse (275–288 nm, 5 ns duration) was prepared by the frequency doubling of the visible output of the dye laser (Scanmate II, Lambda Physik) pumped by the second harmonic (532 nm) of a Nd:YAG laser (Powerlite 8010) through a beta barium borate (BBO) crystal. For velocity map imaging (VMI)^17^,^18^ experiment, the aforementioned first laser pulse (pump) was used to excite the molecule whereas the second UV laser pulse (probe) was employed to ionize the nascent methyl radical *via* its 3P_z_^2^A′′ (*ν* = 0) ← X[combining tilde]^2^A′′ transition of ˙CH_3_ (333.484 nm) or ˙CD_3_ (333.826 nm). The delay time between pump and probe laser pulses was fixed at 25 ns. The polarization of the pump laser pulse was parallel to a detector plate in the VMI experiment. For the two-color slow-electron velocity map imaging (SEVI)^19^,^20^ experiment, the probe (ionization) pulse was delayed by 5 ns with respect to the pump laser pulse. The position-sensitive detector equipped with the dual microchannel plates (MCP; Burle, *φ* = 40 mm) and phosphor screen was used for both VMI and SEVI experiments. A CCD camera (IDS USB 2.0 UI-2230SE-M-GL) was employed to capture the images using the LABVIEW based acquisition program.^21^ Raw images were reconstructed by the MEVELER program.^22^ The wavelength was calibrated by the Wavemaster (Continuum). The VMI and SEVI experimental results were calibrated according to the well-known energetics of O_2_ dissociation dynamics.

## Results and discussion

**Fig. 2:**
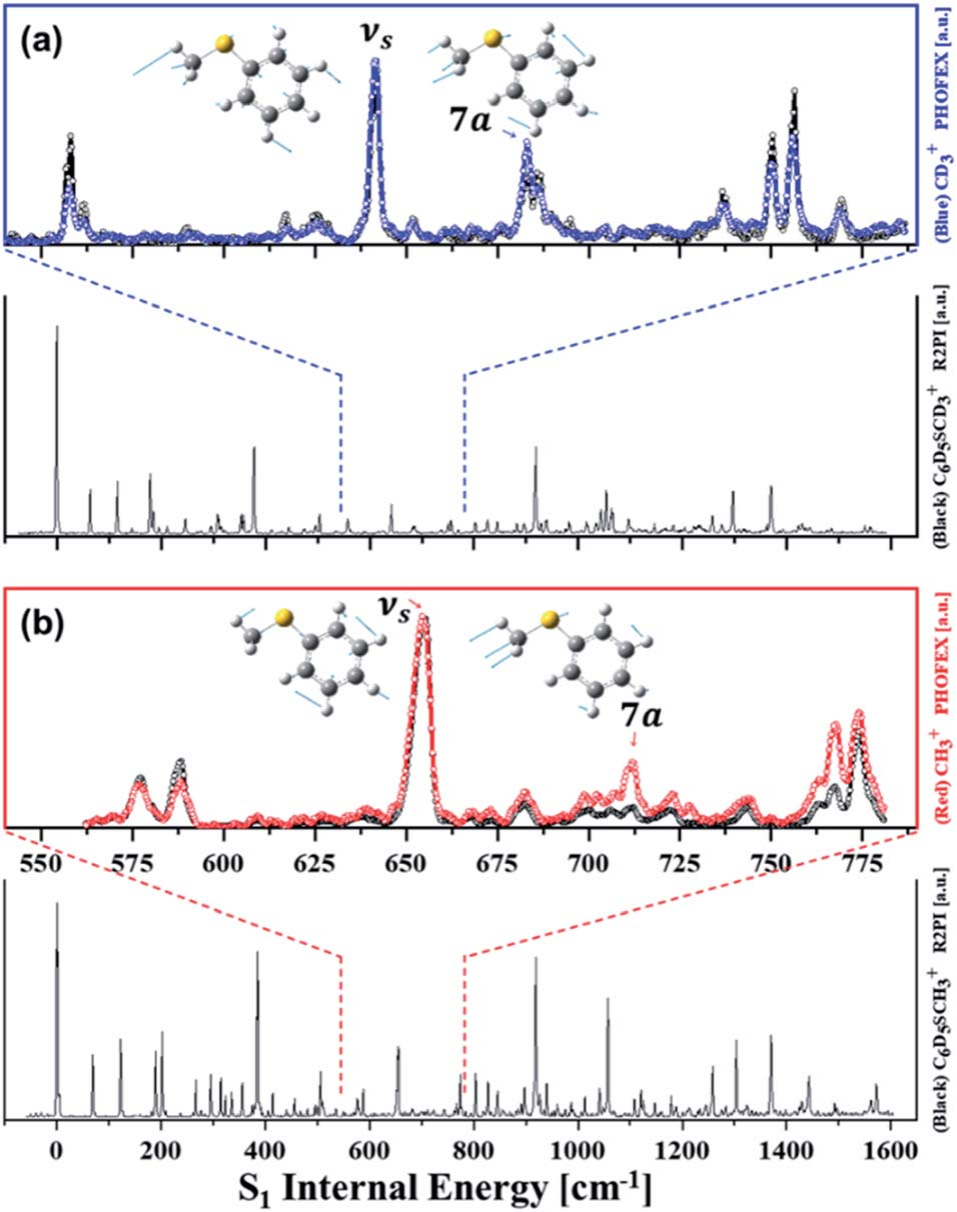
(a) (black) R2PI or (blue) (1 + 1′) PHOFEX spectrum of C_6_D_5_SCD_3_ (b) (black) R2PI or (red) (1 + 1′) PHOFEX spectrum of C_6_D_5_SCH_3_; ν_S_: symmetric S–CH_3_ (or S–CD_3_) stretching mode, 7a: anti-symmetric S–CH_3_ (or S–CD_3_) stretching mode.^3^,^6^,^7^,^23^ Inset images depict S_1_ normal mode displacement vectors calculated by the Gaussian 09 software^24^ using the B3LYP method with the 6-311++G(3df, 3pd) basis set. Detailed mode assignments are summarized in Table S2.†

**Fig. 3:**
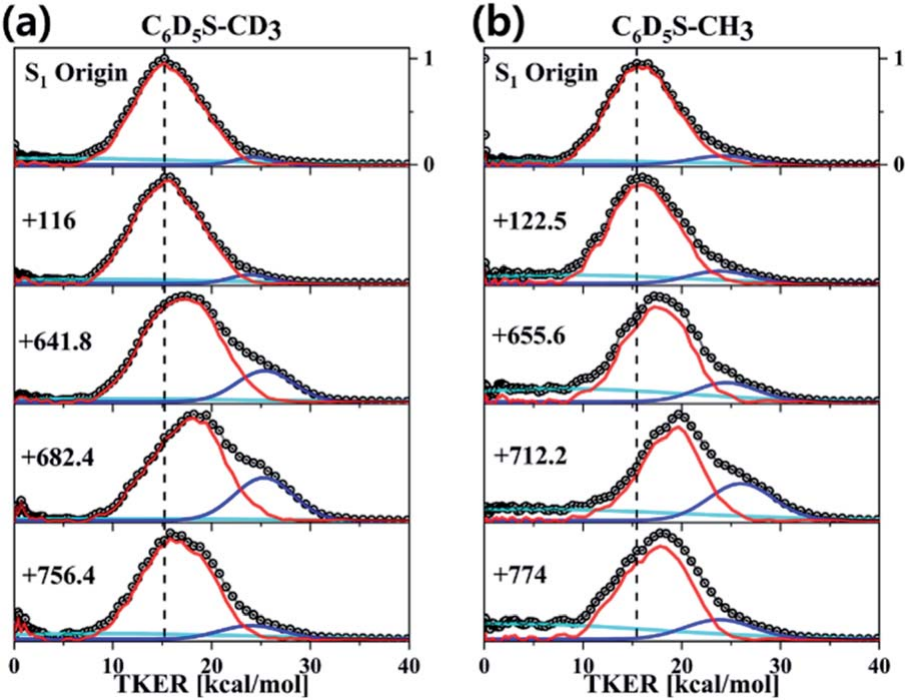
Total kinetic energy release (TKER) distributions obtained by the analyses of the translational energy distributions of the methyl fragment. TKER distributions from (a) C_6_D_5_S–CD_3_ or (b) C_6_D_5_S–CH_3_ at selected S_1_ vibronic modes. Red or blue lines represents the contribution of the reaction channel leading to the Ã or X[combining tilde] products, respectively, while the two-photon background is depicted as the cyan line. Vertical dashed line indicates represent the mean values of the translational energy distributions at the S_1_ origins. The sudden shifts of the translational energy distributions are noticeable at dynamic resonances.

Resonance-enhanced two-photon ionization (R2PI) spectra of C_6_D_5_S–CD_3_ and C_6_D_5_S–CH_3_ show very-well resolved S_1_–S_0_ vibronic structures, Fig. 2. Mode assignments are quite straightforward as those of similar thioanisoles had been already spectroscopically established in earlier studies^3^,^6^,^7^ and also confirmed by SEVI spectroscopies in this work (see the ESI†). Photofragment-excitation (PHOFEX) spectrum taken by probing the nascent CD_3_ or CH_3_ fragment from C_6_D_5_S–CD_3_ or C_6_D_5_S–CH_3_ as a function of the S_1_–S_0_ excitation energy, respectively, is also shown, Fig. 2. While R2PI and PHOFEX spectra of both isotopomers are almost identical in all the energy region explored here, it is quite surprising to observe that the 7a vibronic band of C_6_D_5_S–CH_3_ is nearly absent in R2PI whereas the significant PHOFEX signal is observed at the same mode. We have found that the 7a mode of C_6_D_5_S–CH_3_ shows the strongly enhanced X[combining tilde]/Ã product branching ratio, indicating that the nonadiabatic transition is highly facilitated by the 7a mode excitation (*vide infra*). The experimental fact that the 7a mode is inactive in the R2PI spectrum, therefore, strongly suggests that the corresponding lifetime may be quite shortened. Translational energy distributions obtained by the velocity-map ion images taken from the ionization of nascent CD_3_ or CH_3_ fragment from C_6_D_5_S–CD_3_ and C_6_D_5_S–CH_3_ are shown in Fig. 3, respectively. In most of bands, the X[combining tilde]/Ã product branching ratio remains low, indicating that the predissociation reaction is pretty much adiabatic in nature. On the other hand, it is obvious that the X[combining tilde]/Ã product branching ratio shows resonances at both 7a and ν_S_ modes for C_6_D_5_S–CD_3_, whereas it stands out only for the 7a mode for C_6_D_5_S–CH_3_. The X[combining tilde]/Ã product branching ratio (*Γ*) for C_6_D_5_S–CD_3_ is estimated to be 0.22 ± 0.06 or 0.34 ± 0.05 for the ν_S_ or 7a mode, respectively, compared to the value of 0.04 ± 0.03 for the zero-point energy level. This indicates that both ν_S_ and 7a mode excitations are effective in facilitating the nonadiabatic channel in the S–CD_3_ predissociation process while the latter seems to be more efficient. On the other hand, the X[combining tilde]/Ã product branching ratio is measured to be 0.19 ± 0.02 or 0.43 ± 0.03 at ν_S_ or 7a mode of C_6_D_5_S–CH_3_, respectively, compared to *Γ* ∼ 0.08 ± 0.04 at its S_1_ zero-point level. This experimental finding suggests that the 7a mode excitation of C_6_D_5_S–CH_3_ is much more efficient in expediting the nonadiabatic path compared to ν_S_, Table 1. The main question is then how these data could be interpreted qualitatively or more ideally quantitatively. As the X[combining tilde]/Ã product branching ratio is the consequence from the competition between the nonadiabatic electronic predissociation (Herzberg type-I) and adiabatic vibrational predissociation (Herzberg type-II), it may reflect the extent of the proximity of the corresponding quantum state to the conical intersection seam in terms of energetics and structures. In other words, the larger X[combining tilde]/Ã product branching ratio results from the higher probability of the nonadiabatic electronic predissociation process, and this may be ascribed to that the associated quantum level is closer to the conical intersection seam by any means. In this sense, the experimentally measured X[combining tilde]/Ã branching ratios in all different isotopomers represent the projections of the S_1_/S_2_ conical intersection seam onto different normal mode platforms.

**Table 1:** The vibrational frequencies of ν_S_ and 7a with corresponding X[combining tilde]/Ã branching ratios of thioanisole isotopomers

S_1_ mode	C_6_H_5_S–CH_3_^*a*^	C_6_H_5_S–CD_3_^*b*^	C_6_D_5_S–CH_3_^*c*^	C_6_D_5_S–CD_3_^*c*^
000 (X[combining tilde]/Ã)	34 504 (0.053)	34 516 (0.07)	34 664 (0.081 ± 0.04)	34 677 (0.04 ± 0.030)
ν_S_	+683 (0.165 ± 0.025)	+656 (0.33 ± 0.04)	+656 (0.19 ± 0.02)	+642 (0.22 ± 0.06)
7a	+722 (0.416 ± 0.033)	+705 (0.38 ± 0.04)	+712 (0.43 ± 0.03)	+682 (0.34 ± 0.05)

^*a*^ Ref. 3.

^*b*^ Ref. 6.

^*c*^This work [cm^–1^].

**Fig. 4:**
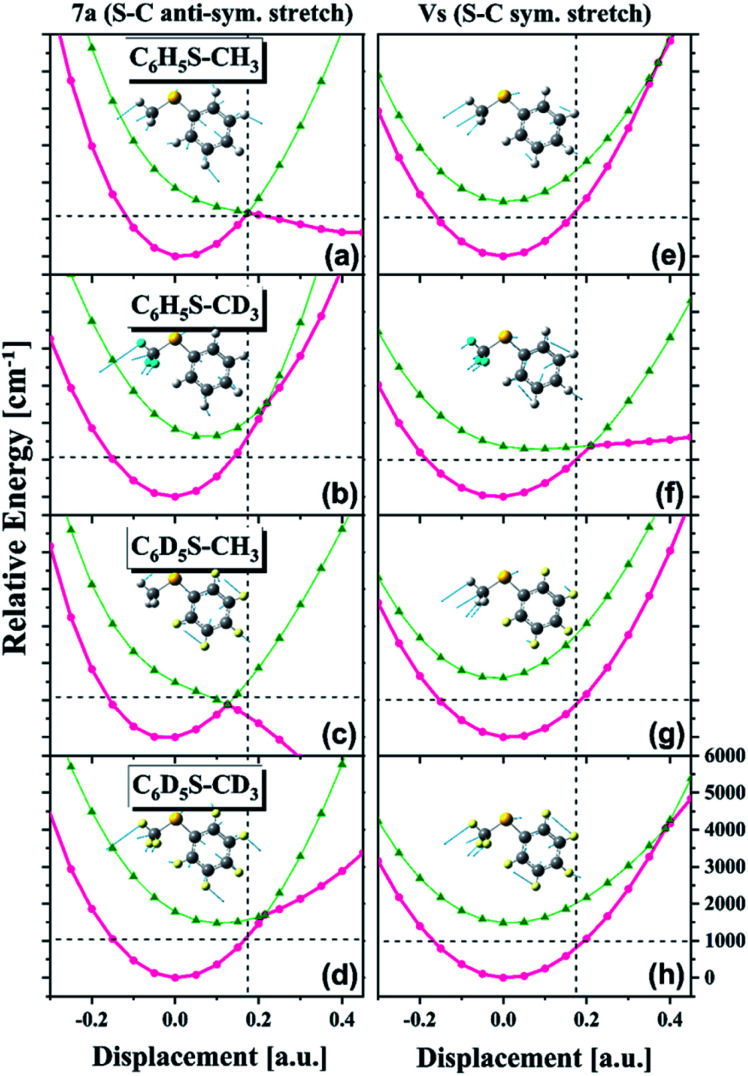
Calculated potential energy curves along (left) 7a and (right) ν_S_ normal modes for four thioanisole isotopomers. Pink and olive lines are supposed to represent adiabatic S_1_ and S_2_ states, respectively. Relative electronic energies (ordinate) are plotted *versus* the nuclear displacement associated with specific normal modes (abscissa). Excited states have been calculated using the TDDFT method with the 6-311++G (3df, 3pd) basis set. Positions in terms of the energy and nuclear displacement of the S_1_/S_2_ curve crossing predicted in the bare thioanisole along the 7a mode are drawn as dotted lines for the comparison.

Obviously, it is nontrivial to depict a multidimensional map of the conical intersection seam from the present experimental results alone although the proximity of the quantum level to the conical intersection seam is certainly correlated with the measured X[combining tilde]/Ã product branching ratio. Instead of trying to describe the conical intersection seam directly, we have carried out theoretical calculations of potential energy surfaces for various isotopomers particularly along the critical modes of ν_S_ or 7a, Fig. 4. First of all, it is very interesting to find out that the S_1_/S_2_ potential energy surfaces along the ν_S_ or 7a normal mode are so different for different isotopomers. Namely, the energetics and gradients associated with curve crossing are strongly influenced by the subtle difference of the normal mode space induced by the combinatorial way of the H/D substitution. It is remarkable that the S_1_/S_2_ curve crossing points calculated along the one-dimensional coordinate are extremely valuable for the interpretation of the experiment as below. It should be noted that the X[combining tilde]/Ã product branching ratios should linearly correlate with the bifurcation ratios of the reactive fluxes prepared near the conical intersection. Therefore, the qualitative comparison of the X[combining tilde]/Ã product branching ratios among various isotopomers based on differences in the S_1_/S_2_ potential energy surface crossings should be still reasonable. For comparison of the experiment with theoretical calculation, we will discuss the results of all four thioanisole isotopomers (C_6_H_5_S–CH_3_, C_6_D_5_S–CH_3_, C_6_H_5_S–CD_3_, and C_6_D_5_S–CD_3_).

The most dramatic shape of the conical intersection, for example, could be inferred from the calculated S_1_/S_2_ curve crossing along the 7a mode of C_6_H_5_S–CH_3_ or C_6_D_5_S–CH_3_, giving the X[combining tilde]/Ã product branching ratio of 0.416 ± 0.033^3^ or 0.43 ± 0.03, respectively (Fig. 4(a) and (c)). In these circumstances, the S_1_/S_2_ cure crossing produces the low-lying repulsive adiabatic curve along the 7a mode extension. This would certainly facilitate the rather fast escape of the reactive flux trapped in the upper adiabatic curve onto the repulsive part of the potential energy curve leading to the predissociation reaction. The exceptionally high X[combining tilde]/Ã product branching ratio at 7a for both C_6_H_5_S–CH_3_ and C_6_D_5_S–CH_3_ indicates that the corresponding reactive flux undergoes the nonadiabatic transition with the significantly high probability. It should be reminded that the nonadiabatic reaction path does not give the X[combining tilde] product exclusively at the asymptotic limit. Rather, it gives rise to both Ã and X[combining tilde] products with the former of the relatively even higher probability in most cases.^5^ In this regard, the probability of bifurcation into the nonadiabatic channel at the 7a mode excitation of C_6_H_5_S–CH_3_ or C_6_D_5_S–CH_3_, relative to the adiabatic channel, should be regarded to be much higher than implied by the experimentally measured X[combining tilde]/Ã product branching ratio, although time-resolved dynamic studies are necessary for the more quantitative argument. The repulsive nature of the S_2_ diabatic curve along the 7a mode particularly stands out for C_6_D_5_S–CH_3_, Fig. 4(c). This may explain the peculiar spectral features observed in R2PI and PHOFEX spectra of C_6_D_5_S–CH_3_ (*vide supra*) in Fig. 2. The experimental finding that the R2PI signal is almost absent at the 7a mode excitation implies that the corresponding lifetime may be exceptionally short as the R2PI signal gets significant strength only when the S_1_ state survives long enough during the laser ionization process by the nanosecond laser pulse. Overall, the S_1_/S_2_ crossing point along the 7a mode is found to be located around 1200 cm^–1^ above the S_1_ minimum energy point for C_6_H_5_S–CH_3_ and C_6_D_5_S–CH_3_, Fig. 4(a) and (c). Considering the zero-point energy of ∼360 cm^–1^ for the 7a mode, this energetic position of the curve crossing point is quite close to the first quanta of the 7a mode, suggesting that the conical intersection is likely to be easily accessed by the 7a mode excitation of those molecules. For all other isotopomers, the S_1_/S_2_ curve crossing points along the 7a mode are predicted to be located at ∼2000 cm^–1^ or less above the S_1_ minimum energy points, Fig. 4(a)–(d). These energetic positions are still close to the first quanta of the 7a mode considering that the calculated are often overestimated. The significantly large X[combining tilde]/Ã product branching ratio of 0.38 ± 0.04 or 0.34 ± 0.05 estimated for the 7a mode excitation of C_6_H_5_S–CD_3_ or C_6_D_5_S–CD_3_, respectively, reflects that the corresponding vibronic excitation prepares the reactive flux at the conical intersection seam along which the S–CD_3_ bond elongation should be effectively coupled. At the present time, it is less straightforward to quantify the structural and energetic characteristics from the comparison of the experimentally measured X[combining tilde]/Ã branching ratio with the calculated potential energy surfaces.

One of the most puzzling experimental observations has been the fact that the X[combining tilde]/Ã product branching ratio shows the strong resonance-like enhancement at the ν_S_ mode excitation of C_6_H_5_S–CD_3_ giving the X[combining tilde]/Ã product branching ratio of 0.33 ± 0.04, whereas the ν_S_ mode excitation is not so effective in facilitating the nonadiabatic transition for C_6_H_5_S–CH_3_ to give *Γ* ∼ 0.165 ± 0.025.^3^,^6^ This has been considered to be peculiar as such a huge effect of the methyl moiety deuteration on the nonadiabatic dynamics is not anticipated. In this regard, it is quite interesting to find out here that the S_1_/S_2_ crossing point is calculated to be more accessible by the one quantum of ν_S_ for C_6_H_5_S–CD_3_, Fig. 4(f), whereas it is less likely to be accessible for the case of C_6_H_5_S–CH_3_, Fig. 4(e). In other words, the S_1_/S_2_ crossing point along ν_S_ is located energetically much higher for C_6_H_5_S–CH_3_ compared to that of C_6_H_5_S–CD_3_ with respect to their S_1_ minima. Moreover, the S_1_/S_2_ crossing point of C_6_H_5_S–CH_3_ is quite remote from the S_1_ minimum energy position in terms of the nuclear displacement compared to that of C_6_H_5_S–CD_3_. It is indeed remarkable that the simple potential curve calculation could explain the experimental finding very well at least qualitatively. The effect of deuteration of the methyl moiety on the reaction nonadiabaticity seems to be similarly applicable to isotopomers of C_6_D_5_S–CH_3_ and C_6_D_5_S–CD_3_ at the ν_S_ mode excitation, giving the X[combining tilde]/Ã branching ratio of 0.19 ± 0.02 and 0.22 ± 0.06, respectively. The S_1_/S_2_ curve crossing points of two isotopomers along ν_S_ support the experiment, as the crossing point of C_6_D_5_S–CD_3_ is predicted to be located lower than that of C_6_D_5_S–CH_3_ with respect to the S_1_ minima of those isotopomers, Fig. 4(g) and (h). And yet, it should be noted that the X[combining tilde]/Ã branching ratio generally increases slowly with increasing the energy with the different slopes depending on the molecular characteristics.^3^ In this sense, the X[combining tilde]/Ã branching ratios estimated at the ν_S_ mode excitations of C_6_H_5_S–CH_3_ and C_6_D_5_S–CH_3_ are hardly associated with dynamic resonances in the proximity of the conical intersection seam, and thus the explanation of those experimental results based on the potential energy curve crossings seems to be less meaningful at the present time. The dynamic role of the pseudo-conical intersection previously predicted to be existed at the C–C–S–CH_3_ dihedral angle of 90°^25^ has not been considered in this work, while it could be important in the high S_1_ internal energy region where the out-of-plane modes are likely to be more activated.

## Conclusions

Quantum mechanical characterization of the multidimensional conical intersection seam has been demonstrated to be feasible in predissociation reaction of the excited thioanisole molecule. The projection of the S_1_/S_2_ conical intersection seam onto the normal mode space could be inferred from the nature of the S_1_/S_2_ vibronic modes responsible for dynamic resonances showing the large enhancement of the nonadiabatic transition probability. The X[combining tilde]/Ã product branching ratios estimated from the translational energy distributions of the nascent methyl fragments measured for various isotopomers of thioanisole are particularly useful as the proximity of the initial reactive flux to the conical intersection seam could be judged from different viewpoints set by the different normal mode space. Accordingly, the nonadiabatic bifurcation dynamics of the reactive flux into either the Herzberg type-I (electronic) or type-II (vibrational) predissociation pathway has been disentangled and the structure and dynamic role of the conical intersection seam could be inspected from various points of view. With the aid of simple calculations of potential energy curves along selected normal mode displacements, we report the first cornerstone for the otherwise formidable characterization of the conical intersection seam in polyatomic molecular system.

## Conflicts of interest

There are no conflicts to declare.

## Supplementary Material

Supplementary information
