# Supplementary material for: Multidimensional characterization of the conical intersection seam in the normal mode space
Source: Chem Sci. 2020 Jun 16;11(26):6856–61. doi: 10.1039/d0sc02045a (PMC7504900; doi:10.1039/d0sc02045a)
Supplement: Supplementary file 1 [file SC-011-D0SC02045A-s001.pdf]

Electronic Supplementary Material (ESI) for Chemical Science.
This journal is © The Royal Society of Chemistry 2020

Supplementary Information

Multidimensional Characterization of the Conical Intersection Seam in the Normal Mode Space

Heesung Lee, †So-Yeon Kim, and Sang Kyu Kim*

Department of Chemistry, KAIST, Daejeon 34141, Republic of Korea

*Corresponding Author: sangkyukim@kaist.ac.kr

Present Addresses: † LG Chem, R&D campus, Daejeon 34122, Republic of Korea

Table of Contents

SI1. SEVI (slow electron velocity map imaging) spectra via selected S_1 vibrations

SI1-1. Ionization potentials for thioanisole isotopomers

SI1-2. Symmetric (ν_s) and anti-symmetric (7a) S-C stretching vibrations

SI1-3. Vibrational mode assignments

SI2. Deconvolution of TKER distributions of two thioanisole isotopomers

SI2-1. Two-component ($\tilde{A}$, $\tilde{X}$) manual deconvolution

SI2-2. Three-component ($\tilde{A}_1$, $\tilde{A}_2$, $\tilde{X}$) gaussian function-aided deconvolution

SI3. Sample information (NMR, Chromatography)

SI1. SEVI (slow electron velocity map imaging) spectra via selected S_1 vibrations

SI1-1. Ionization potentials for thioanisole isotopomers

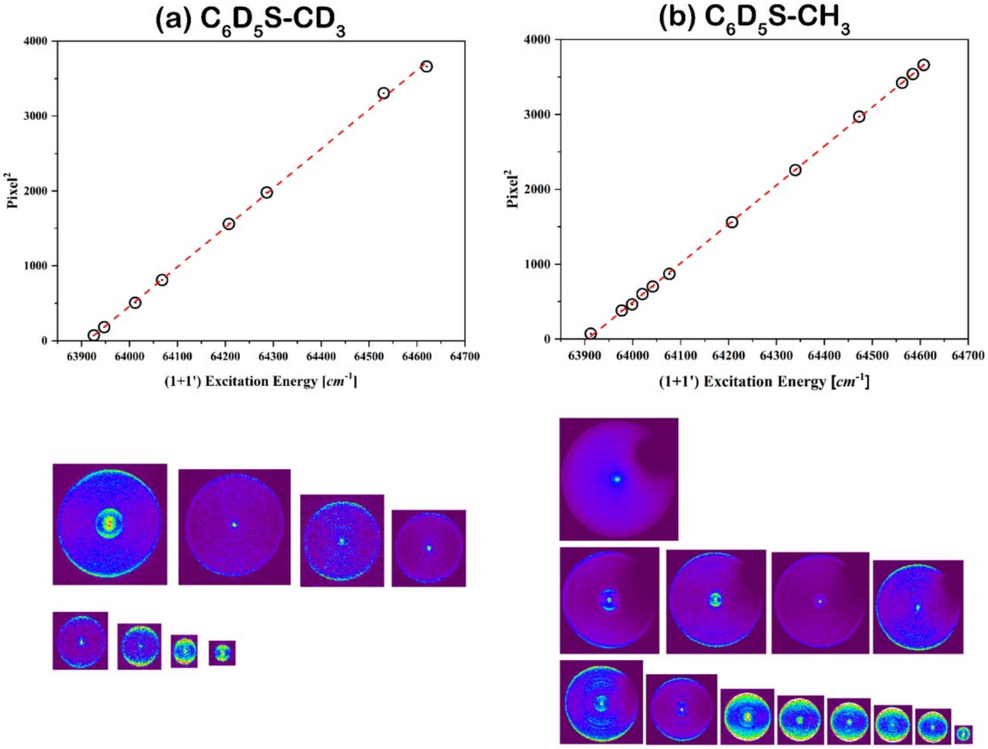

Figure S1. Determination of ionization potentials of two thioanisole isotopomers (upper). 2D images (lower) are shown.

For conducting SEVI experiments (Figure S2), two-color threshold-ionization via each 0_0^0 transition of two isotopomers was done as Figure S1. Ionization potential of each isotopomer is derived from the extrapolation of the measured radial displacement of the ring in VMI images with respect to the two-photon input energies. Ionization potentials of all six thioanisole isotopomers are summarized in Table S1.

Table S1. Experimental S_1 origins, and D_0 origins (ionization potential)

	‡C_6H_5S-CH_3	‡ C_6H_5S-CD_3	‡ C_6H_5S-CH_2D_1	‡ C_6H_5S-CH_1D_2	C_6D_5S-CH_3	C_6D_5S-CD_3
**S_1 origin [cm^{-1}]**	34 504	34 516	34 504, 34 510	34 510, 34 516	34 664	34 677
**D_0 origin [cm^{-1}]**	63 899	63 917	63 904, 63 915	63 909, 63 919	63 896	63 902

‡ Supporting Ref. 1 ‡ There are two isomers for each partially deuterated Thioanisole-h_2d_1 and Thioanisole-h_1d_2, respectively.

SI1-2. Symmetric (ν_s) and anti-symmetric (7a) S-C stretching vibrations

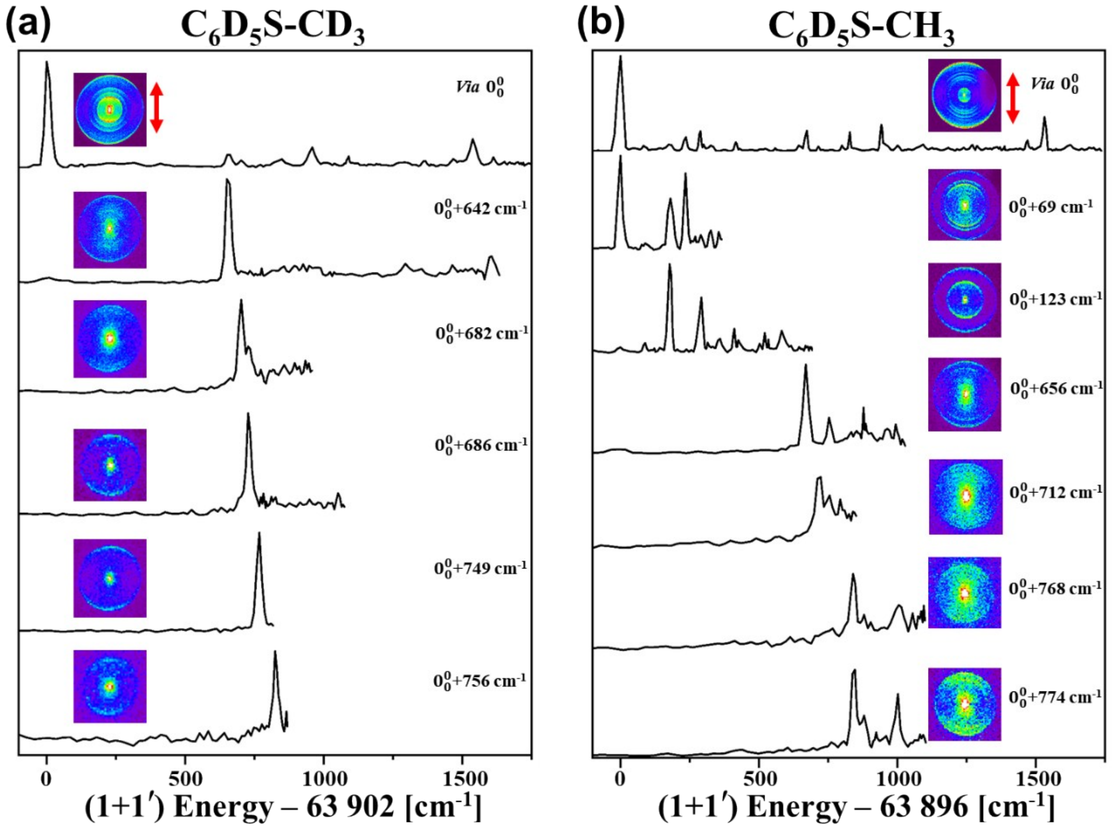

Figure S2. 2 color (1+1′) SEVI spectra via selected S_1 vibronic states of (a) C_6D_5S-CD_3 and (b) C_6D_5S-CH_3. Insets show 2D images having comparable ring radius for the peak in each SEVI spectrum whereas the actual reconstruction of images was carried by MEVELER program[S2] and plotted. Linear polarization axis is shown as red arrow.

S_1 ν_s and 7a modes of two isotopomers are assigned by aids of previous reference values[S3-S6] with SEVI experiments (Figure S2) in this work. Although definitive assignment of the full S_1 normal modes could not be achieved because of limited numbers of SEVI spectra via full S_1 vibronic bands, there are already excellent spectroscopic works for thioanisole molecules.[S3-S6] and authors did reliable assignment for the S_1 ν_s and 7a modes for both C_6D_5S-CD_3 and C_6D_5S-CH_3 by getting SEVI spectra. Subsequent comparisons of SEVI peaks (D_0-S_1 peaks) with calculated values (TDDFT) as well as R2PI peaks engenders the reliable assignments keeping in mind the assumption that the vibronic selection rule ($\Delta\nu = 0$) between D_0 and S_1 is obeyed. Detailed numbers are summarized in Table S2.

SI1-3. Vibrational mode assignments

Table S2. Comparison of calculation and experiment. Normal modes are labelled according to the Varsanyi notation. S_0, D_0, S_1 vibrational energies [cm^{-1}] are calculated using the *Gaussian09* package. The (TD) DFT B3LYP level with 6-311++G(3df,3pd) basis set was used. Assignments are done by comparing previous thioanisole references[S3-S6] with the data of this work (R2PI, TDDFT calculation, and SEVI).

**$C_6D_5S\text{-}CH_3$ [cm^{-1}]**						**$C_6D_5S\text{-}CD_3$ [cm^{-1}]**					
**mode**	**S_0**	**D_0**	**S_1**	**R2PI**	**SEVI**	**mode**	**S_0**	**D_0**	**S_1**	**R2PI**	**SEVI**
τ	48	77	-59		90	τ	43	68	-55		
10b	159	185	124	123	177	10b	139	107	80		
15	191	199	191			15	176	183	176		
τCH_3	228	147	124			τCD_3	186	107	90		
β_s	322	326	325			β_s	309	313	312		
16a	359	341	485			16a	358	341			
6a	412	404	410			6a	405	395			
16b	424	386	427			16b	424	386			
6b	605	578				6b	605	578	524		
4	553	515				4	553	515	485		
v_s	670	660	674	656	669	v_s	656	642	658	642	651
7a	724	689	720	712	711	7a	691	674	693	682	702
11						$v_s\tau^1$			709	686	727
10a						10a					
$\gamma_s CH_3$	972	924	913			$\gamma_s CD_3$	734	700	688		
17b	760	857	623			17b	760	788	623		
12	957	955	938			12	957	955	937	918	
$\beta_{as} CH_3$	984	981	962			$\beta_{as} CD_3$	773	767	764		
17a	803	827				17a	803	827			
1						1					
5	827	857				5	827	857			
18a						18a					
18b						18b					
9b	856	862				9b	856	862			
9a	885	896	862			9a	885	896	862		
14						14			834		
$\beta_s CH_3$	1358	1362	1347			$\beta_s CD_3$	1032	1034	1025		
3						3					
$\beta_s CH_2$	1486	1455	1467			$\beta_s CD_2$	1076	1046	1056		
19b	842	852	802	768	840	19b	842	855	804		
$\gamma_{as} CH_3$	1469	1459				$\gamma_{as} CD_3$	1062	1054	1049		

※ 1→9a, 9b↔14 (assignment change from Supporting Ref. 3,4 to this study)

SI2. Deconvolution of TKER distributions of two thioanisole isotopomers

SI2-1. Two-component ($\tilde{A}$,$\tilde{X}$) manual deconvolution

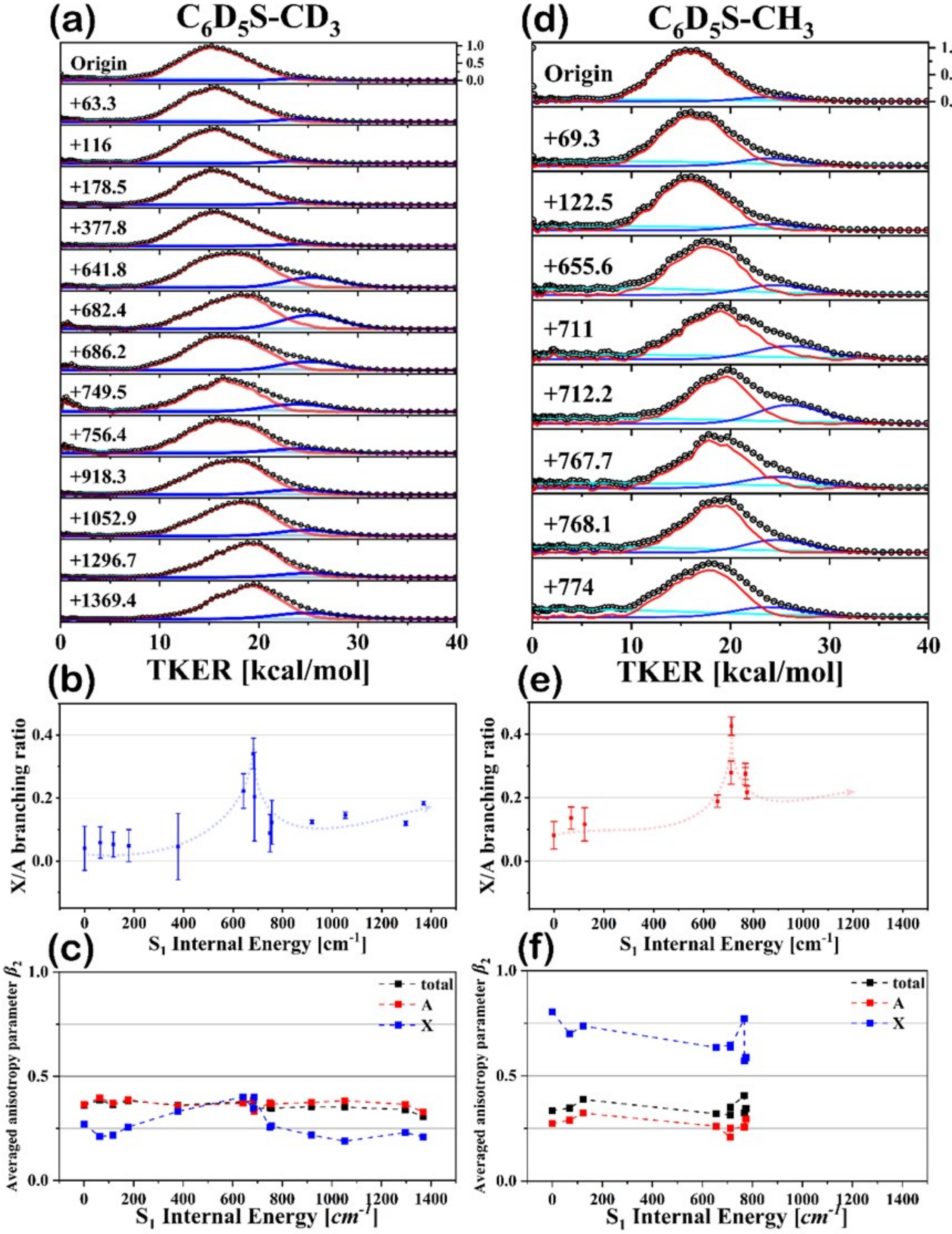

Figure S3. (a), (d) full translational energy (total kinetic energy release; TKER) distributions. TKER distributions of (a) C_6D_5S-CD_3 and (d) C_6D_5S-CH_3 via selected S_1 vibronic transitions followed by fragmentated into (a) $\cdot CD_3$ and (d) $\cdot CH_3$ detected by (2+1) REMPI via their $3P_z$ state, respectively. Ordinate: normalized population, black-hollow circle: experiment, red: manually fitted $\tilde{A}$ component, blue: gaussian fitted $\tilde{X}$ component, emerald: gaussian fitted two-photon background. Sum of fitted two components ($\tilde{A}$ and $\tilde{X}$) is drawn in black solid line. $\tilde{X}/\tilde{A}$ branching ratios of (b) C_6D_5S-CD_3 and (e) C_6D_5S-CH_3 and averaged anisotropy parameters (β_2) of (c) C_6D_5S-CD_3 and (f) C_6D_5S-CH_3 are shown.

Authors first deconvoluted multiphoton backgrounds from the reconstructed TKER distributions. Considering estimated maximum available TKER for $\tilde{A}$ and $\tilde{X}$ products,[S8] most evident $\tilde{X}$ component in TKER distribution via 7a mode was fitted as a single gaussian function. The rest of that TKER distribution was assumed as $\tilde{A}$ products. Authors call this as 'manual deconvolution'. Whereas, to calculate uncertainties, TKER distributions are fitted as a sum of two individual gaussian functions. Error bars for $\tilde{X}/\tilde{A}$ were then calculated from the $\pm 1\sigma$ fitting errors of these two Gaussian functions. Based on the above 'manual' deconvolution concept, corresponding averaged anisotropy parameters were calculated for two components as well as totally (full range of TKER) averaged anisotropies with respect to the S_1 internal energy, Figure S3 (c) and (f). Contrast features of $\tilde{X}$ components of two isotopomers could be recognized. This infers that the C_6D_5S-CH_3 dissociates faster then the C_6D_5S-CD_3 does. Wider vibronic band widths of C_6D_5S-CH_3 than the corresponding narrower widths of C_6D_5S-CD_3 parent R2PI and fragment PHOFEX in Figure 2 strongly support this interpretation.

SI2-2. Three-component ($\tilde{A}_1$, $\tilde{A}_2$, $\tilde{X}$) gaussian function-aided deconvolution

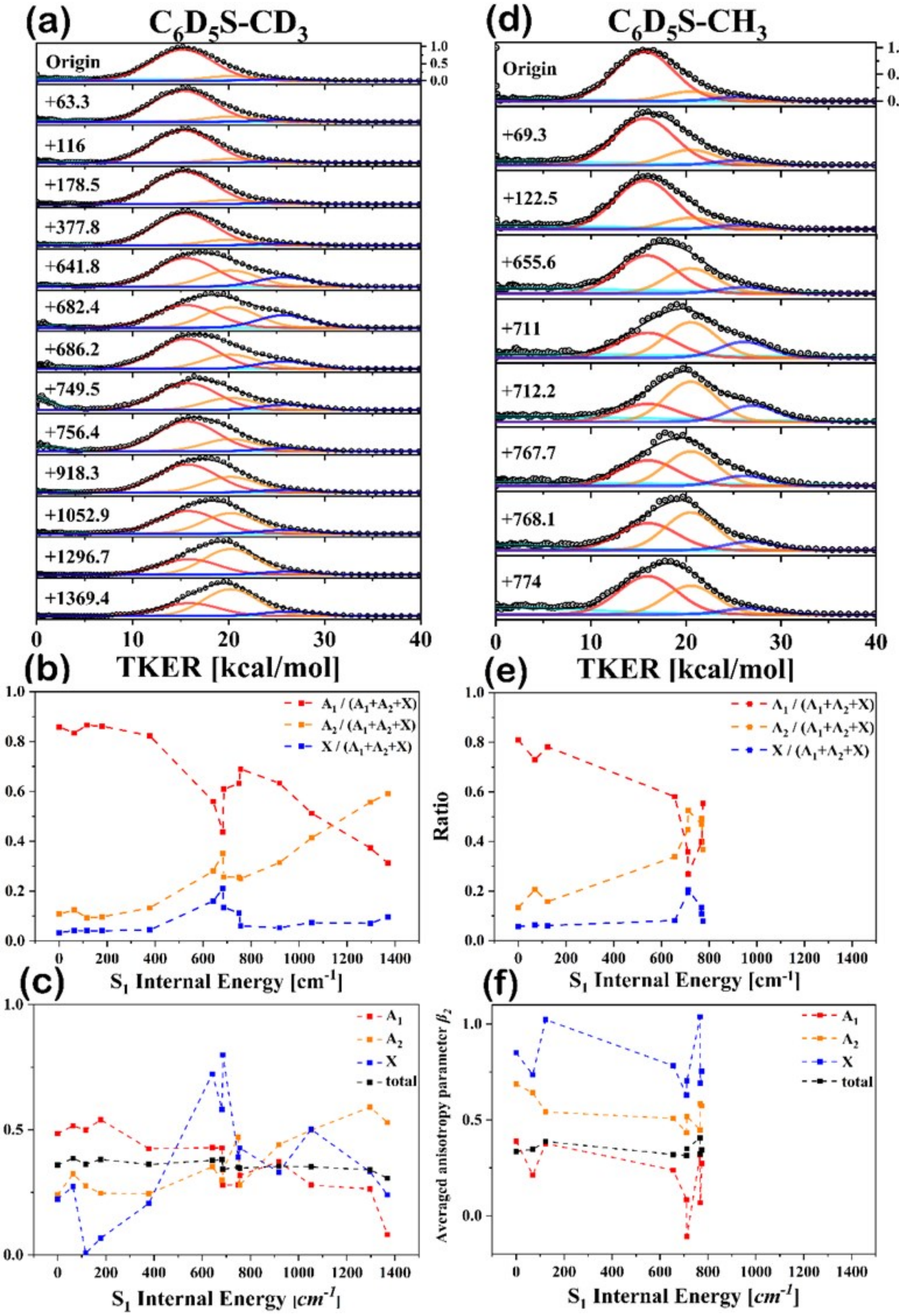

Figure S4. (a), (d) full translational energy (total kinetic energy release; TKER) distributions. TKER distributions (upper) of (a) $C_6D_5S\text{-}CD_3$ and (d) $C_6D_5S\text{-}CH_3$ via S_1 vibronic transitions followed by fragmentated into (a) $\cdot CD_3$ and (d) $\cdot CH_3$ detected by (2+1) REMPI via their $3P_z$ state, respectively. Ordinate: normalized population, black-hollow circle: experiment, red: gaussian fitted $\tilde{A}_1$ component, orange: gaussian fitted $\tilde{A}_2$ component, blue: gaussian fitted $\tilde{X}$ component, emerald: two-photon background. Sum of simulated three components ($\tilde{A}_1$, $\tilde{A}_2$, and $\tilde{X}$) is drawn in black solid line. Branching ratios of (b) $C_6D_5S\text{-}CD_3$ and (e) $C_6D_5S\text{-}CH_3$ and averaged anisotropy parameters (β_2) of (c) $C_6D_5S\text{-}CD_3$ and (f) $C_6D_5S\text{-}CH_3$ are shown.

From the previously-reported time-resolved work, it has been found that the bifurcation starting from the S_1/S_2 conical intersection ends up with two distinct reaction pathways. These pathways are very different in terms of its X/A product branching ratio as well as the translational energy distribution of the A state. Although the bifurcation ratio should be linearly proportional to the X/A product branching ratio (as above in (5)), they are quantitatively different. This is because that one channel (vibrational predissociation) gives rise to the A state almost exclusively whereas the other channel (electronic predissociation) produces both A and X products. In the former case of vibrational predissociation, the A state is internally highly excited, giving the low translational energy distribution (A_1). On the other hand, the A state from the electronic predissociation is much less excited internally, giving the relatively higher kinetic energy distribution (A_2). Based on these experimental facts, the fit to the experiment is given to extract the bifurcation ratios instead of the final product X/A branching ratios. However, since the current experimental results are not time-resolved, the deconvolution is intrinsically ambiguous to give large uncertainties during the fit.

SI3. Sample information (NMR, Chromatography)

$C_6D_5S\text{-}CH_3$

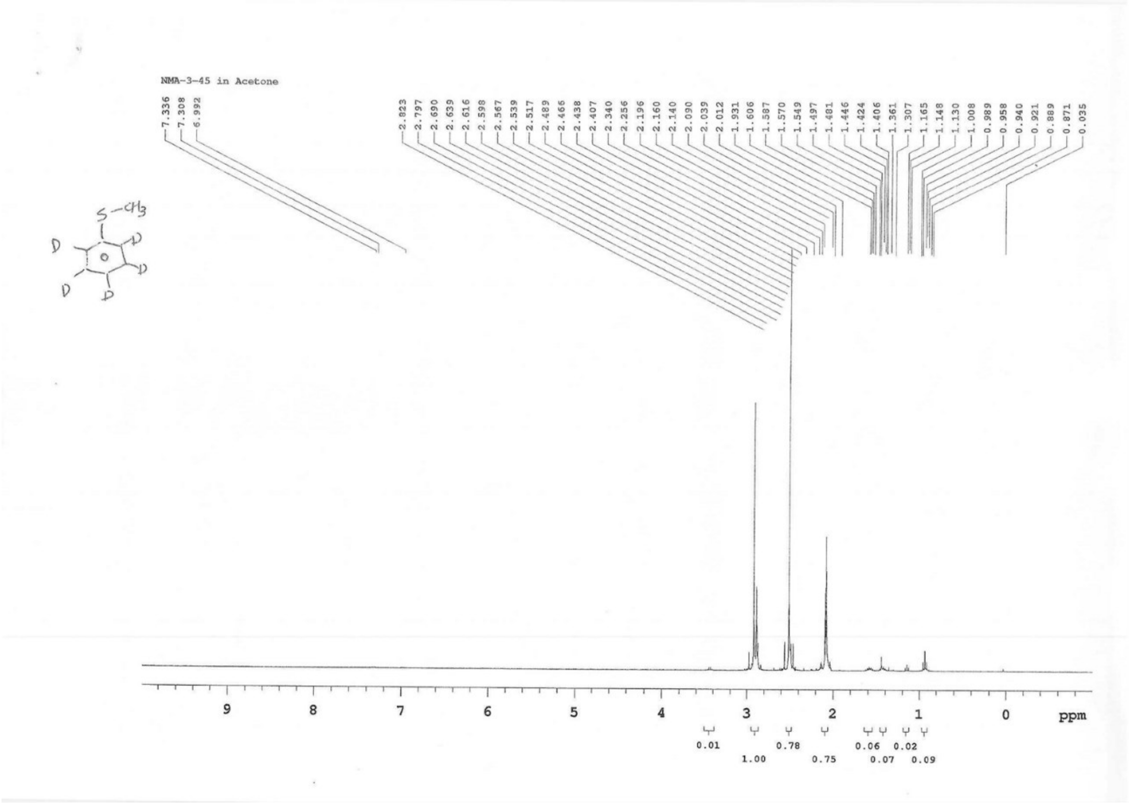

Chromatogram and Results

MEDIGEN

Injection Details

Injection Name:	2-NMA-3-45	*Run Time (min):*	20.00
Vial Number:	2	*Injection Volume:*	20.00
Injection Type:	Unknown	*Channel:*	UV_VIS_1
Calibration Level:		*Wavelength:*	254.0
Instrument Method:	ACN100%-20min-0.5	*Bandwidth:*	n.a.
Processing Method:	ACN100%-20min-1.0	*Dilution Factor:*	1.0000
Injection Date/Time:	11-Oct-16 16:56	*Sample Weight:*	1.0000
Column type:	Thermo Acclaim 120 C18 5μ*4.6mm*150mm		

Chromatogram

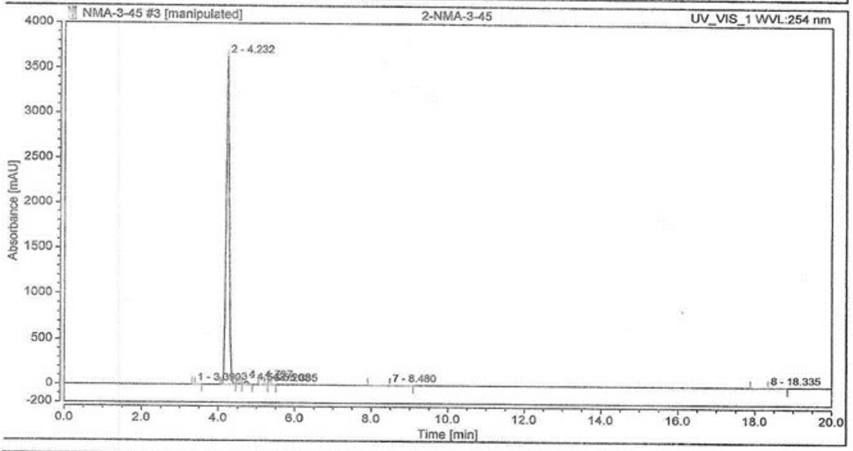

Integration Results

No.	Peak Name	Retention Time min	Area mAU*min	Height mAU	Relative Area %	Relative Height %	Amount n.a.
1		3.390	0.185	2.789	0.05	0.08	n.a.
2		4.232	338.462	3631.141	98.50	98.68	n.a.
3		4.542	0.541	6.858	0.16	0.19	n.a.
4		4.737	3.443	33.598	1.00	0.91	n.a.
5		5.205	0.264	2.638	0.08	0.07	n.a.
6		5.385	0.122	1.218	0.04	0.03	n.a.
7		8.480	0.278	0.681	0.08	0.02	n.a.
8		18.335	0.324	0.866	0.09	0.02	n.a.
Total:			343.620	3679.788	100.00	100.00	

C_6D_5S-CD_3

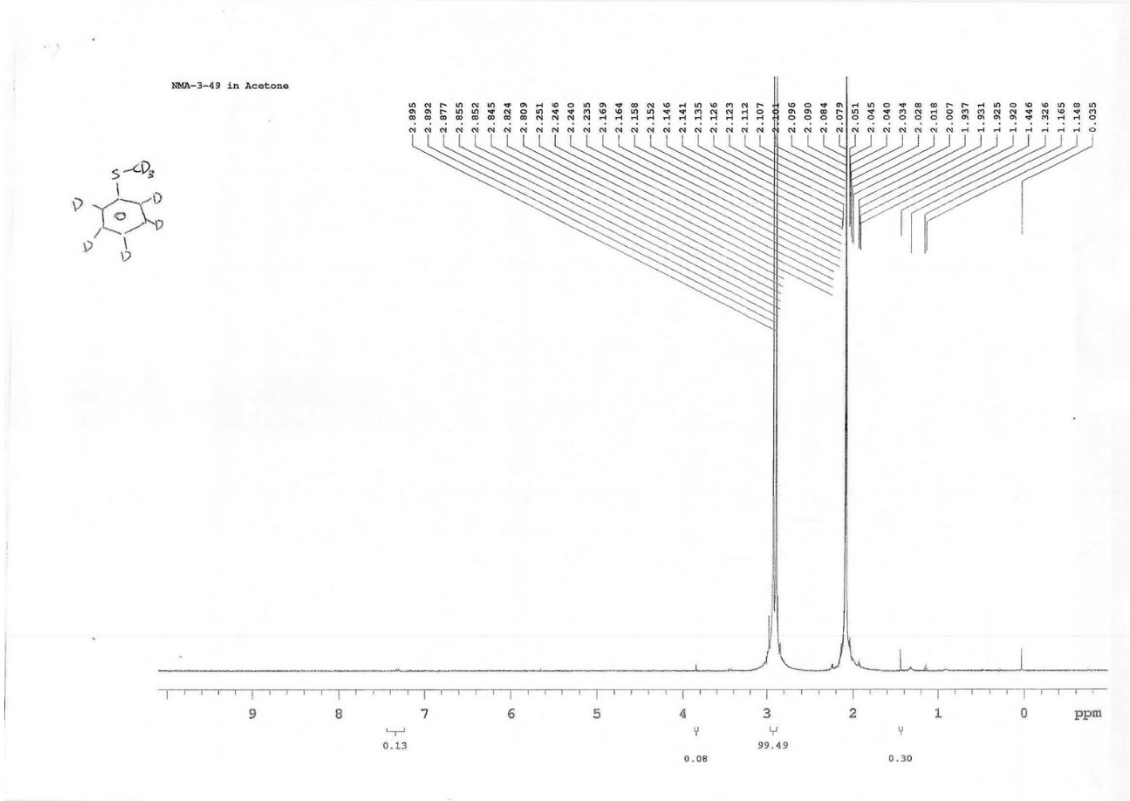

NMA-3-49 in Acetone
2.895
2.892
2.877
2.855
2.852
2.845
2.824
2.809
2.251
2.246
2.240
2.235
2.169
2.164
2.158
2.152
2.146
2.141
2.135
2.126
2.123
2.112
2.107
2.101
2.096
2.090
2.084
2.079
2.051
2.045
2.040
2.034
2.028
2.018
2.007
1.937
1.931
1.925
1.920
1.446
1.326
1.165
1.148
0.035
9
8
7
6
5
4
3
2
1
0
ppm
0.13
0.08
99.49
0.30

Chromatogram and Results

MEDIGEN

Injection Details

Injection Name:	2-NMA-3-49-1	*Run Time (min):*	20.00
Vial Number:	2	*Injection Volume:*	20.00
Injection Type:	Unknown	*Channel:*	UV_VIS_1
Calibration Level:		*Wavelength:*	254.0
Instrument Method:	ACN100%-20min-0.5	*Bandwidth:*	n.a.
Processing Method:	ACN100%-20min-1.0	*Dilution Factor:*	1.0000
Injection Date/Time:	14-Oct-16 15:59	*Sample Weight:*	1.0000
Column type:	Thermo Acclaim 120 C18 5μ*4.6mm*150mm		

Chromatogram

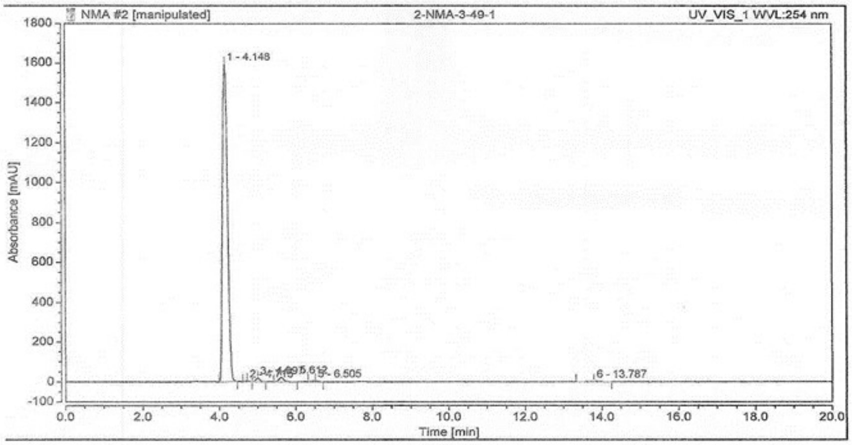

Integration Results

No.	Peak Name	Retention Time min	Area mAU*min	Height mAU	Relative Area %	Relative Height %	Amount n.a.
1		4.148	246.213	1593.205	97.17	96.80	n.a.
2		4.715	0.450	4.154	0.18	0.25	n.a.
3		4.997	2.315	19.295	0.91	1.17	n.a.
4		5.612	3.099	22.988	1.22	1.40	n.a.
5		6.505	0.552	3.724	0.22	0.23	n.a.
6		13.787	0.757	2.439	0.30	0.15	n.a.
Total:			253.386	1645.805	100.00	100.00	

Supplementary References

1. Lee, J.; Kim, S.-Y.; Kim, S. K., Spectroscopic Separation of the Methyl Internal-Rotational Isomers of Thioanisole Isotopomers (C_6H_5S-CH_2D and C_6H_5S-CHD_2). *The Journal of Physical Chemistry A* **2014**, 118 (10), 1850-1857.

2. Dick, B., Inverting ion images without Abel inversion: maximum entropy reconstruction of velocity maps. *Phys. Chem. Chem. Phys.* **2014,** *16* (2), 570-580.

3. Lim, J. S.; Kim, S. K., Experimental probing of conical intersection dynamics in the photodissociation of thioanisole. *Nat. Chem.* **2010**, 2 (8), 627-632.

4. Lee, J.; Kim, S.-Y.; Kim, S. K., Spectroscopic Separation of the Methyl Internal-Rotational Isomers of Thioanisole Isotopomers (C_6H_5S-CH_2D and C_6H_5S-CHD_2). *The Journal of Physical Chemistry A* **2014**, 118 (10), 1850-1857.

5. Han, S.; Lim, J. S.; Yoon, J.-H.; Lee, J.; Kim, S.-Y.; Kim, S. K., Conical intersection seam and bound resonances embedded in continuum observed in the photodissociation of thioanisole-d3. *The Journal of Chemical Physics* **2014**, 140 (5).

6. Kim, S.-Y.; Lee, J.; Kim, S. K., Conformer specific nonadiabatic reaction dynamics in the photodissociation of partially deuterated thioanisoles (C_6H_5S-CH_2D and C_6H_5S-CHD_2). *Phys. Chem. Chem. Phys.* **2017,** *19* (29), 18902-18912.

7. Cederbaum, L. S.; Friedman, R. S.; Ryaboy, V. M.; Moiseyev, N., Conical Intersections and Bound Molecular States Embedded in the Continuum. *Physical Review Letters* **2003**, 90 (1), 013001.

8. Kim, J. B.; Yacovitch, T. I.; Hock, C.; Neumark, D. M., Slow photoelectron velocity-map imaging spectroscopy of the phenoxide and thiophenoxide anions. *Phys. Chem. Chem. Phys.* **2011,** *13* (38), 17378-17383.

9. Lim, J. S.; You, H. S.; Kim, S.-Y.; Kim, S. K., Experimental observation of nonadiabatic bifurcation dynamics at resonances in the continuum. *Chemical Science* **2019,** *10* (8), 2404-2412.

10. G. M. Roberts, D. J. Hadden, L. T. Bergendahl, A. M. Wenge, S. J. Harris, T. N. V. Karsili, M. N. R. Ashfold, M. J. Paterson and V. G. Stavros, *Chemical Science*, **2013**, (*4*), 993-1001.
